# Single-beat global atrial mapping facilitates the treatment of short-lived atrial tachycardias and infrequent premature atrial contractions

**DOI:** 10.1007/s10840-022-01405-8

**Published:** 2022-10-25

**Authors:** Rita B. Gagyi, Anna M. E. Noten, Krista Lesina, Bakhtawar K. Mahmoodi, Sing-Chien Yap, Mark G. Hoogendijk, Sip Wijchers, Rohit E. Bhagwandien, Tamas Szili-Torok

**Affiliations:** Department of Cardiology, Electrophysiology, Erasmus MC, University Medical Center Rotterdam, 2040, 3000 Rotterdam, Netherlands

**Keywords:** Brief episodes of atrial tachycardia, Premature atrial complex, Catheter ablation, Mapping

## Abstract

**Background:**

Short runs of atrial tachycardias (ATs) and infrequent premature atrial contractions (PACs) are difficult to map and ablate using sequential electrophysiology mapping techniques. The AcQMap mapping system allows for highly accurate mapping of a single atrial activation.

**Objectives:**

We aimed to test the value of a novel dipole charge density-based high-resolution mapping technique (AcQMap) in the treatment of brief episodes of ATs and PACs.

**Methods:**

Data of all patients undergoing catheter ablation (CA) using the AcQMap mapping system were reviewed.

**Results:**

Thirty-one out of 219 patients (male *n* = 8; female *n* = 23) had short runs of ATs (*n* = 23) and PACs (*n* = 8). The mean procedural time was 155.3 ± 46.6 min, with a mean radiation dose of 92.0 (IQR 37.0–121.0) mGy. Total radiofrequency application duration 504.0 (271.0–906.0) s. Left atrial localization of ATs and PACs was identified in 45.1% of the cases, right atrium localization in 45.1%, and septal origins in 9.8% of the cases. Acute success was achieved in 30/31 (96.8%), and recurrence during the follow-up developed in six patients (19.4%), including four patients with PACs and two patients with short-lived ATs. One patient presented procedure-related groin hematoma as minor complication.

**Conclusion:**

Brief episodes of highly symptomatic ATs and infrequent PACs can be mapped using charge density mapping and successfully ablated with high acute and long-term success rates.

**Supplementary Information:**

The online version contains supplementary material available at 10.1007/s10840-022-01405-8.

## Introduction

Atrial tachycardias (AT) represent an important cause of supraventricular tachycardias (SVT) accounting for approximately 3 to 17% of all SVTs [1, 2]. Catheter ablation (CA) is considered a standard approach in AT therapy [1]. Compared to AVNRT and AVRT ablations in which there is a well-defined electrophysiological endpoint, during CA of ATs ablation, the arrhythmias need to be induced and mapped in order to perform successful CA [3, 4]. Frequently, patients present with brief episodes of ATs or premature atrial contractions (PACs) that are difficult to induce during electrophysiology (EP) study. Patients with symptomatic PACs frequently show inadequate number of PACs for mapping during the electrophysiology procedures. In the majority of these cases, the patients are either not accepted for ablation, the attempt is unsuccessful, or the patients more frequently develop recurrences after a CA attempt [5].

A novel dipole charge density-based mapping modality provides the possibility of global atrial mapping which hypothetically eliminates most of the current limitations [6]. The AcQMap imaging and mapping system rapidly creates highly accurate ultrasound based-cardiac chamber 3D reconstruction combined with high-resolution charge density-based maps of electrical activation, which permits the mapping of any cardiac rhythm pattern with stable and variable cycle length (CL) or even a single atrial beat [7, 8]. Preliminary results on using the AcQMap system for patients with short runs of ATs showed high clinical success rates of this approach [9]. In this study, we aim to demonstrate the value of this novel mapping technique in a larger cohort of patients with extended follow-up.

## Methods

### Study design and primary hypothesis

In this single-center retrospective study, we investigated outcomes of CA procedures in patients presented with highly symptomatic short runs of ATs or PACs using a novel mapping modality (AcQMap, Acutus Medical Inc., Carlsbad, CA). We included patients with brief episodes of ATs and PACs referred for single-beat mapping due to the inability to use standard sequential mapping techniques. We hypothesized that short runs of ATs and PACs can be mapped and successfully ablated with the use of the novel dipole charge density-based noncontact mapping system.

### Endpoints

The primary endpoint of this study was defined as procedural efficacy measured by acute procedural success. The secondary endpoints were long-term outcome defined as number of recurrences, and time to ablation therapy measured by months from first diagnosis.

### Patient selection

A number of 219 consecutive patients with SVTs referred for CA utilizing the AcQMap mapping system were screened. The inclusion period was between 24/07/2017 and 24/11/2021. We excluded patients in whom the EP study revealed atrial fibrillation and sustained mappable AT and included only patients with brief episodes of ATs and PACs in the final analysis. In patients with ATs: the arrhythmia duration was ranging from 3 to 124 beats documented on 24–72-h Holter monitoring. During the procedures, AT duration was measured after induction or spontaneous onset, we did not cumulatively determine AT duration. Induction of AT was performed with programmed atrial stimulation with or without isoproterenol administration, up to 3 atrial extrasystoles. Based on their medical history, two groups of patients were identified in this study: a group of patients who were referred for redo procedure due to frequent recurrences after the previous procedure(s) (pulmonary vein isolation and/or atrial tachycardia ablation), and a group of *de novo* patients. The *de novo* patient group consisted of patients who had non-sustained atrial tachyarrhythmias and were specifically planned for EP study and atrial mapping using AcQMap with a possible CA procedure. Patients who were previously denied from CA procedure because the documented arrhythmia episodes were considered too short for mapping were also included in the *de novo* patient group.

### Data collection

The institutional medical ethics committee approved the data collection for this study and concluded that it did not fall under the Medical Research Involving Human Subjects Act (ACUTUS Registry MEC-2018–1640). Baseline demographic and clinical characteristics from patients with brief episodes of ATs and PACs were collected using the electronic health records. Procedural data were derived both from the electronic medical files, as well as from the electronic procedural log files recorded by the AcQMap system.

The following demographic and procedural data were collected from the patients: sex, year of birth, age at procedure, height, weight, BMI, date of procedure, procedure duration time, number of applications, application duration, fluoroscopy time, acute success, and procedure-related complications. Acute procedural success was defined as the absence of tachyarrhythmias during a waiting time 15–30 min after the last ablation application. Major complications were defined as any procedure-related adverse event, which were life-threatening, required significant surgical intervention and prolonged hospital stay, or resulted in death. Minor complications were defined as procedure-related adverse events, which resulted in minimal transient impairment of a body function or damage to a body structure, or which did not require any intervention or further therapy.

Furthermore, we collected AcQMap mapping data: number of AcQMaps performed, mapping time, chamber origin, location, and mechanism of ATs. In addition, we collected and analyzed clinical data, such as left atrial dimension, left ventricular function, comorbidities, and antiarrhythmic medication.

### Procedures

The CA procedures were performed under either local or general anesthesia. Vascular access was obtained with femoral venous puncture, and multipolar diagnostic catheters were placed within the heart: the coronary sinus, the right ventricle, and the region of the bundle of His. A special dual-purpose introducer sheath (AcQRef) with integrated electrodes was introduced. The AcQRef provides stable electrical reference and eliminates the need for an additional introducer and quadripolar reference catheter. Diagnostic EP study was performed in every case according to standard EP test. In redo patients, reconduction of the pulmonary veins (PVs) was checked, and re-isolation was performed if needed. We reconstructed the endocardial anatomic surface and then overlaid high-resolution charge density maps of electrical activation using the AcQMap high-resolution imaging and mapping system. Ablation was performed using either the Celsius ThermoCool (Biosense Webster, US) catheter or MagnoFlush (Medfact, Germany) ablation catheter at discretion of the operator. Intravenous heparin was administered for anticoagulation, guided by the activated clotting time (> 350 s for LA and > 300 s for RA). We interpreted propagation history maps, identified atrial activation patterns, and performed targeted radiofrequency (RF) catheter ablation.

### Mapping technique

**Fig. 1:**
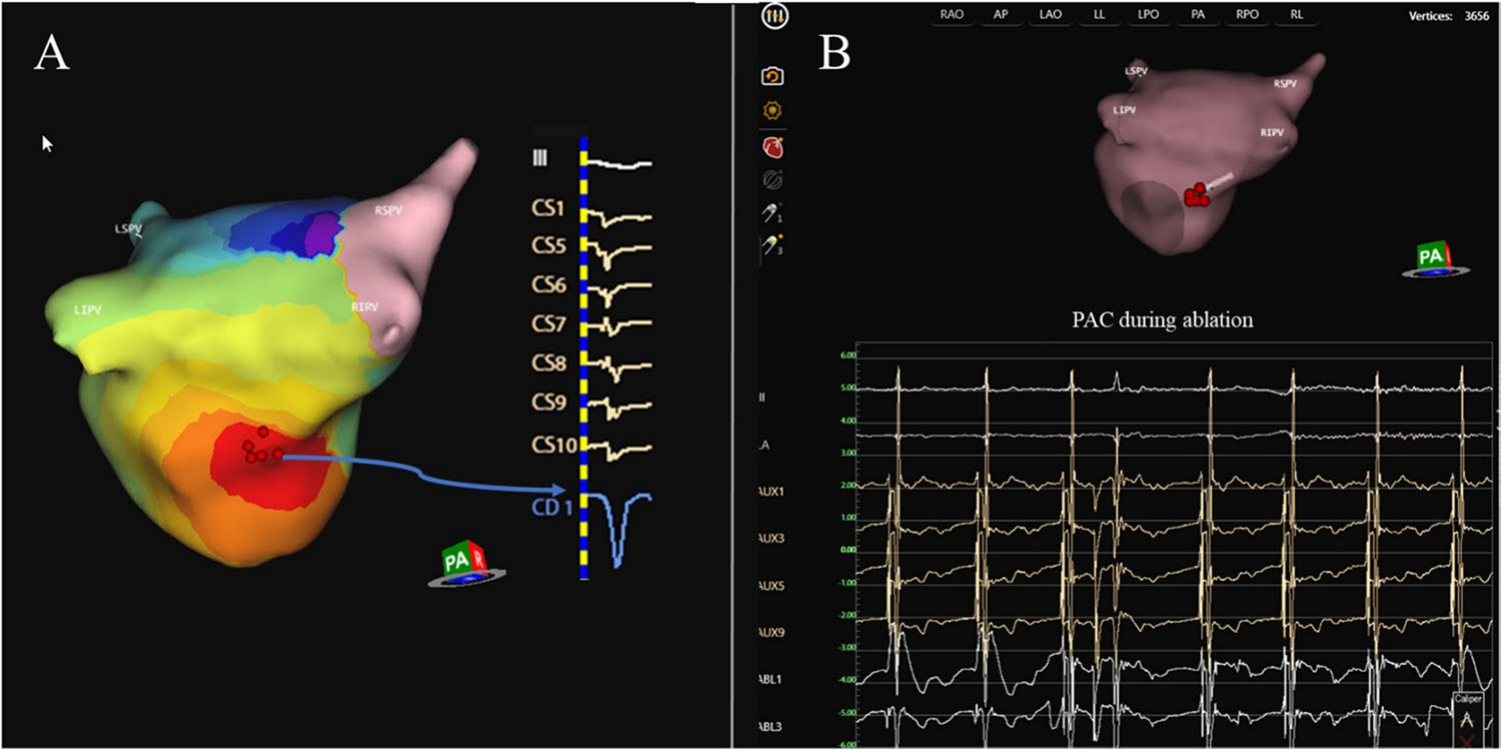
Premature atrial complex ablation originating in the left atrium. Example of a patient presenting with premature atrial complex originating in the left atrium (LA). In panel **A**, a PA view of the LA representing the propagation history maps with breakout on the inferior posterior wall. The color red is used to indicate the leading edge of the wave front with the trailing color bands showing earlier locations of the wave front. The activation wave front continues spreading up the posterior wall and further down the inferior wall. CD1 represents the local charge density signal at the breakout point with a QS morphology. CS activation and lead III are also represented. Ablation sites on the AcQMap anatomy shell and intracardiac electrograms during ablation are shown in panel **B** as red dots

**Fig. 2:**
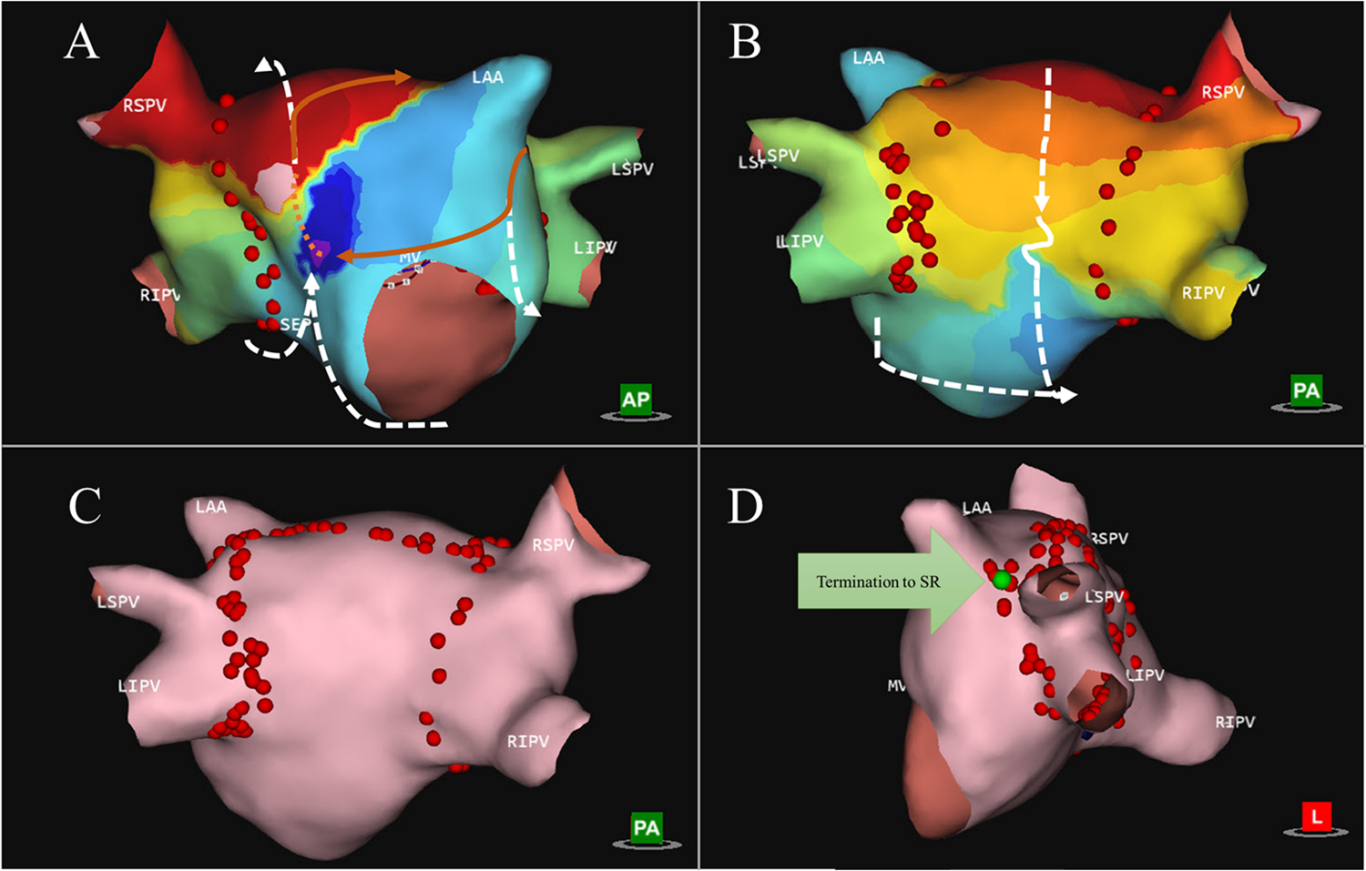
Short-lasting atrial tachycardia with macro-reentry mechanism. The main driving circuit is a macro-reentry around the left atrial appendage with double loop bystander. Panel **A** shows propagation history map of bystander 1, which propagates from the anterior wall, down the ridge around LIPV to the posterior-inferior side, underneath RIPV and up the septum again to the anterior wall (arrows showing the direction of propagation). Panel **B** shows propagation history map of bystander 2 which starts around the RPVs, down the posterior side, squeezing through the slow zone, in a CCW rotation underneath the RIPV and up the septum (see arrows). Panel **C** shows ablation map, where a roofline was performed and extension over the ridge (red dots). Panel **D** shows the site during ablation where termination to sinus rhythm was achieved (green arrow)

The AcQMap high-resolution imaging and mapping system provides maps of electrical activation across an ultrasound-acquired cardiac chamber surface and localizes supplementary electrode catheters within and around the surface. The diagnostic recording AcQMap 10F catheter (AcQMap; Acutus Medical) has a 25-mm diameter spheroid-shape formed by six splines, with each containing eight ultrasound transducers interspersed between eight biopotential electrodes. This catheter is introduced into the chamber of interest over a 0.032-inch guidewire. The catheter and system are designed to reconstruct 3D endocardial anatomy from reflection points marked by ultrasound transducers without the need to contact the chamber surface. In order to ensure the entire endocardial surface is sampled, the user continuously rotates the catheter approximately 60° in both directions during ultrasound point acquisition. The system samples up to 115,000 surface points per minute. The entire set of surface points are usually collected within 2–3 min. An impedance-based localization system tracks the position of the electrodes, integrates ultrasound distances, and registers the acquired surface mash. The resulting chamber anatomy corresponds to the end-diastolic size and shape. The AcQMap catheter has 48 biopotential electrodes measuring the non-contact unipolar voltage field. The unipolar voltages together with the endocardial anatomy are key inputs for the inverse solution to derive the location of charge sources on the endocardial surface. The inverse solution is based on Poisson’s equation, which defines how the potential field at any point (measured as voltage) is equal to the local sources plus the sum of distant sources. Activation maps are created within 2 min and displayed as a spatiotemporal window of activation history across the reconstructed 3D image. A propagation-history map uses bands of color to show the location and velocity of the leading edge of the wave front over a set duration of time (Figs. 1 and 2).

The AcQMap system comprises of two different non-contact mapping modalities, single-position mapping and SuperMap. In this study, we mostly utilized the single-position mapping because it can be applied as single-beat analysis to map short non-sustained AT or PACs. For single-position maps, the AcQMap catheter needs to be centrally positioned within the chamber of interest.

### Catheter ablation and follow-up

Ablation was performed using the abovementioned catheters with a power setting between 10 and 50 W and temperature limit set to a maximum of 43 °C with flow rate of 17–30 ml/min using the Stereotaxis remote magnetic navigation system (Stereotaxis, St. Louis, MO). The system can be integrated with the AcQMap 3D mapping system, and it permits the use of several irrigated and non-irrigated catheters from different brands.

Following the ablation procedure, all patients were monitored, and checked at the outpatient clinic at regular intervals. Follow-up visits were planned based on institutional methodology plan. In patients with ATs and PACs as the primary indication for CA, a follow-up visit at 3 months after the procedure was planned. In the cases where there was no recurrence at the follow-up visit, the patients were discharged. In the post-PVI related AT patient group, follow-up visits were planned at 3, 6, and 12 months after the procedure as part of our standard follow-up protocol. Seven-day Holter monitoring was performed at every follow-up visit.

### Statistical analysis and data analysis

Statistical analysis was performed using SPSS software version 25. Mean and standard deviation (SD) were calculated for normally distributed continuous variables. Median and interquartile range (IQR) were computed for continuous variables with non-normal distribution. Categorical data was presented as absolute numbers and percentages. To compare procedural data between the patient groups, we used independent *T* test. To compare not normally distributed data between patient groups, we used independent *t* test and Mann–Whitney *U* test. One-sample *T* test was used to compare procedural efficiency data to historical control. Due to the limited consistent mapping times available in the literature, historical data were obtained from the German catheter ablation registry [10, 11].

## Results

### Demographic and baseline clinical data

A total number of 219 patients were planned for a mapping procedure utilizing the Acutus 3D mapping system. In 150 patients, an AcQMap was performed during the procedure. In the remaining patients, either arrhythmia was non-inducible or PVI only strategy was followed for CA of AF without complex mapping. From the mapped patients, 71 had a diagnosis of atrial fibrillation (AF), and 48 patients had long lasting AT (> 5 min). We identified 23 patients (male *n* = 6; female *n* = 17) with brief episodes of highly symptomatic ATs, and eight patients with premature atrial contractions (PACs) (male *n* = 2; female *n* = 6). The median arrhythmia duration was 6.5 (IQR 3.75–24.5) episodes. Eighteen patients had a repeat procedure (redo patient group *n* = 18); 13 patients had a *de novo* procedure (*de novo* patient group *n* = 13). None of the redo patients had previous attempt using the Acutus mapping system. The mean age of patients was 51.6 ± 16.4 years. Patient demographic and clinical data are summarized in Table 1.

**Table 1:** Baseline characteristics

	Total	Redo	*De novo*	*p* value
Number of patients (*n*) (proportions %)	31 (100%)	18 (58.1%)	13 (41.9%)	
Age, years	51.6 ± 16.4	52.7 ± 16.1	50.0 ± 17.2	0.65
Female	23 (74.1%)	10 (55.6%)	13 (100.0%)	
Height	171.9 ± 8.3	173.6 ± 8.8	169.6 ± 7.4	0.19
Weight	78.9 ± 12.3	80.2 ± 11.3	77.0 ± 13.8	0.48
BMI	26.7 ± 4.1	26.8 ± 4.1	26.7 ± 4.2	0.49
Antiarrhythmic drug therapy	67.8%	64.7%	81.8%	
LA dimension (mm)	38.1 ± 5.8	39.0 ± 6.0	36.6 ± 5.7	0.45
LAVI	31.1 ± 11.4	33.9 ± 14.0	27.4 ± 7.7	0.50
EF (%)	58.8 ± 5.6	58.2 ± 5.0	59.5 ± 6.3	0.62

Values are presented as mean ± SD

*SD*, standard deviation; *BMI*, body mass index; *LA*, left atrium; *EF*, ejection fraction

### Procedural data

**Fig. 3:**
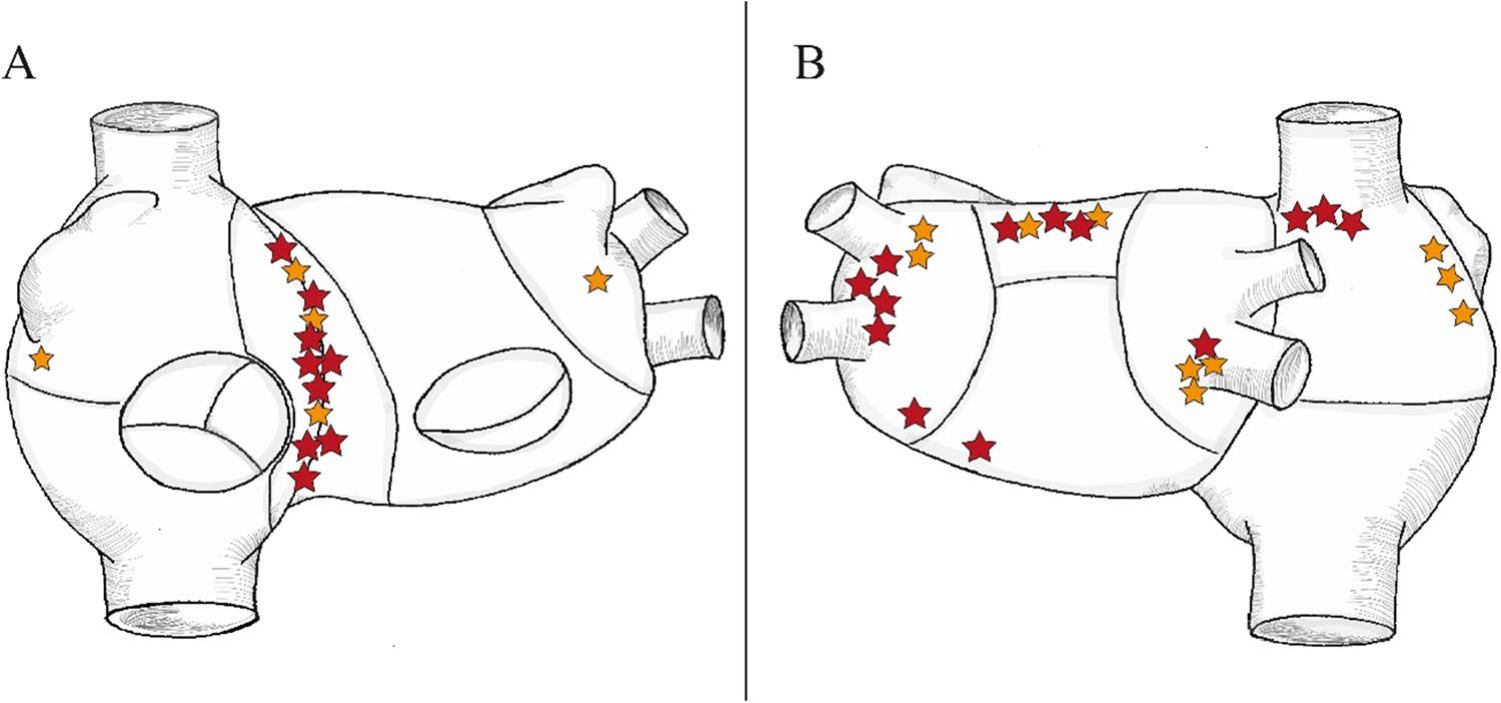
Localization of short ATs and PACs. The sites of ATs mapped with the AcQMap system are shown as yellow (*de novo* AT) and red (redo AT) stars. In panel **A**, the anterior view of the right and left atrium is showed; in panel **B**, the posterior view is visible. Localizations include left atrial (LA) roof (*n* = 5), septal origin (*n* = 10), parahisian origin (*n* = 2), left superior pulmonary vein (LSPV) (*n* = 3), left inferior pulmonary vein (LIPV) (*n* = 1) superior vena cava (SVC) (*n* = 3), carina between LSPV and LIPV (*n* = 2), infero-posterior LA (*n* = 1), lateral wall of the right atrium (RA) (*n* = 3), right inferior pulmonary vein (RIPV) (*n* = 4), between Vein of Marshall (VOM) and left atrial appendage (LAA) (*n* = 1), posterolateral LA (*n* = 1), and right atrial appendage (RAA) basis (*n* = 1)

The mean procedural time was 155.3 ± 46.6 min (mean ± SD), with a mean radiation time 171 8 ± 9.4 min, and radiation dose of 92.0 (IQR 37.0–121.0) mGy. The total mapping time was 192 ± 155 s. Total radiofrequency application duration was 504.0 (IQR 271.0–906.0) s. In the redo group, the total RF application duration was 587.0 (IQR 294.5–965.7) s.; in the *de novo* group, it was 429.0 (194.0–1189.0) s. In patients with PACs, the average procedure duration was 158.3 ± 40.9 min, the average application number was 13.9 ± 9.4, and median fluoroscopy dose was 41.0 (IQR 21.8–113.8) mGy. Procedural data for all patient groups are summarized in Table 2. Regarding the chamber of origin, we found a left atrial (LA) localization of ATs in 45.1% of the cases, right atrium (RA) localization in 45.1% of the cases, and interatrial septal origin requiring RF applications in both LA and RA in 9.7% of the cases. We identified 37 ATs in 31 patients. A more detailed description on localization of arrhythmias is illustrated on Fig. 3. In the PAC patient group, there was one patient presenting PACs from two different locations: inferior to the posterior wall and near LAA basis. Both of these locations were targeted for ablation and ablated successfully. An average number of 2.9 of high-resolution charge density maps were performed in the LA and an average number of 4.4 in the RA. When septal origin was identified, charge density maps were acquired both in the RA and in LA. In 24/31 (77.4%) of the cases, a focal activation pattern was identified on the AcQMaps as target of ablation (Fig. 1 and Video 1). Five patients showed macro/micro-reentry activation patterns (Fig. 2 and Video 2).

**Table 2:** Procedural data

	Total	Redo	*De novo*	*p* value
Total procedure time (min)	155.3 ± 46.6	157.9 ± 46.1	151.7 ± 48.9	0.72
Total application duration (s)	504.0 (271.0–906.0)	587.0 (294.5–965.7	429.0 (194.0–1189.0)	0.54
Total fluoroscopy time (min)	17.1 ± 9.4	19.6 ± 8.2	14.7 ± 10.9	0.08
Total mapping time (s)	192.0 ± 155.0	205.1 ± 162.6	223.8 ± 158.2	0.78
Number of applications	16.2 ± 11.9	17.2 ± 10.7	14.1 ± 14.0	0.58
DAP (median, IQR)	7112.0 (3269.4–8834.8)	8148.4 (5904.4–18,563.6)	6215.6 (2554.3–8797.3)	0.19
X-ray dose (mGy) (median, IQR)	92.0 (37.0–121.0)	96.0 (71.0–223.5)	54.0 (27.4–101.0)	0.05
Acute success (%)	96.8	94.4	100.0	
Average time passed from diagnosis to final procedure (months)	36.1 (9.5–48)	29.0 (16.5–78)	11.0 (6.0–11.0)	0.006

Values are presented as mean ± SD

*SD*, standard deviation; *DAP*, dose area product; *IQR*, interquartile range

### Efficacy and safety data

Acute procedural success was achieved in 30/31 (96.8%) of the cases (22/23 in patients with short-lived ATs, 8/8 in patients with PACs). In one patient, the ablation procedure was unsuccessful because of parahisian location of a perinodal re-entry circuit, where only limited amount of RF energy was applied. Flecainide and beta-blocker medication was initiated in this patient in order to maintain SR. In this case, no recurrence of AT was documented during the follow-up.

Comparing our data to historical data from the German ablation registry, we found no difference in total procedure duration (155.3 ± 46.6 min vs. 157.4 ± 33.6 min; *p* = 0.80), and no difference in total radiation use (17.1 ± 9.4 min vs. 16.9 ± 7.6 min; *p* = 0.93). Using AcQMap, mapping times were much shorter (3.2 ± 2.3 min vs. 15.2 ± 7.6 min; *p* < 0.01). This is approximately five times shorter as compared to previous reported data.

One patient presented procedure-related groin hematoma as minor complication.

### Follow-up data

During the 12-month follow-up period, six patients had documented recurrence (19.4%), including four patients with PACs and two patients with short runs of ATs. One patient had a redo procedure with the AcQMap system 4 months after the index procedure and is recurrence-free since then. Analyzing P-wave morphology in the six patients with recurrence, we found that four patients had ATs with different localization than the initial AT. Two patients showed similar localization of AT on Holter recordings to the localization of the initial arrhythmia. Two additional patients experienced palpitations post-procedurally, but no recurrence of AT was documented. Moreover, Holter continuous rhythm monitoring showed sinus rhythm during symptoms in both patients.

## Discussion

The major finding of the present study is that brief episodes of highly symptomatic ATs and PACs can be mapped using single-position single-beat dipole density charge mapping and ablated successfully with low recurrence rates during follow-up. Although, at first sight, our patient population seems to be small, it is very important to recognize that we describe successful ablation in a group of patients that was previously considered untreatable. Most of the time these patients were either not scheduled for ablation, or even when they underwent diagnostic EP study, due to the lack of appropriate map, they did not undergo catheter ablation. This was a major source of frustration for both the patients and physician.

A very interesting aspect of the current topic is that there is limited literature focused on this specific population. We do believe this problem is underreported; in fact, we could not find any available literature data about successful ablative therapy of brief episodes of ATs or PACs. When comparing our methods and results to those of previous studies, it must be pointed out that all studies included only patients with sustained, long episodes of ATs, and patients with short lasting arrhythmias were not even considered for treatment. Therefore, our results can be interpreted as the first in man systematic report of an interventional approach for patient suffering from brief episodes of ATs and PACs.

### Epidemiology of atrial tachycardias

Based on recent literature, the prevalence of SVT in general population is 2.25 per 1000 persons with a female predominance of 2:1 across all age groups [1]. As part of this group, AT represents approximately 15% of all SVTs, and it may result in remarkably disturbing symptoms in patients. As is well known, in terms of duration, AT may present with brief episodes or sometimes with long stable episodes including incessant ATs. It is by now generally accepted that documentation of the AT and evaluation of the underlying cardiac conditions are fundamental in the diagnosis and management of these arrhythmias [12]. CA is the treatment of choice for symptomatic or recurrent long episodes ATs [13]. Although, the acute procedural success for ATs is high, significant number of the patients with documented ATs develop recurrences. Consequently, many of these cases require a redo procedure within 3 years [14, 15].

### Non-contact vs. contact mapping

In the last decades, remarkable technological improvements provided tools for better mapping of complex arrhythmias, which led to the introduction of electroanatomical mapping systems (EAMS) into clinical practice. Nowadays, the three most widely used contact-based EAMSs are CARTO (Biosense Webster Inc., Irvine, CA, USA), the EnSite X (Abbott, Chicago, IL, USA) and the Rhythmia EAM (Boston Scientific, Marlborough, MA, USA) systems [16, 17]. The main disadvantage of contact-based EAMSs lies in the lack of spatiotemporal stability in the mapping system. While contact-based systems allow visualization of the contact area between the catheter and the cardiac tissue, they cannot provide a precise propagation history of electrical activation in a global view. Contact-based EAMSs have been highly successful in ablating ectopic atrial tachycardia rapidly and with a small number of RF applications [18], but this mapping method is only applicable in relatively long lasting frequently recurrent tachyarrhythmias. Because contact-based EAMS requires manual point-by-point mapping to acquire a proper activation map of the arrhythmia, the mapping of brief episodes is insufficient or even not feasible.

### The evolution of non-contact mapping

To the best of our knowledge, the EnSite multielectrode array (EA) mapping system was the first noncontact mapping technology which emerged in the early stages of 3D mapping, both in terms of technology and concept in mapping [19]. It is notable that this system visualizes the beat-to-beat virtual activation of cardiac arrhythmias, similar to the AcQMap mapping system. It uses a mapping catheter with 64 unipolar electrodes and calculates endocardial unipolar electrograms with the use of a mathematical inverse algorithm. The calculated electrograms are displayed on a surface mesh with 2592 rectangular facets, which represents the geometry of the endocardial surface [19]. It is important to highlight the fact that EA mapping system does not incorporate a true anatomical 3D map, which is a major a disadvantage regarding the temporospatial stability of the system.

Although, the EA mapping technique achieves a success rate of 85% and a long-term success rate of 76% in inducible, sustained AT ablations, it has several additional limitations [19, 20]. It is not consistently used for catheter ablation, and the difficulties of manipulating ablation catheter with the balloon mapping catheter in the atria may have restricted the universal use of this technology [7]. A major source of limitation is due to the lack of endocardial anatomy map, which drives to possible errors in identifying the exact location and mechanism of the arrhythmia. In a study conducted by Wieczorek et al., the EA mapping system was used to guide the ablation of non-sustained focal right atrial tachycardias with promising initial results. From the nine included patients, eight remained AT-free over the follow-up period (16 ± 11 months), but one patient required redo ablation [21]. Large, multicenter randomized studies would be necessary to support these findings; however, because of the above mentioned limitations, it is very unlikely for such studies to be performed.

Compared to the EnSite Array mapping system, the AcQMap mapping system has a significant advantage by offering a global endocardial 3D anatomical map combined with high-resolution charge density maps of electrical activation. Accordingly, AcQMap technology provides the possibility of mapping any cardiac arrhythmia with stable and variable CL, including short-lasting ATs, in real time. This could be the reason why we had much higher success rates even in a patient population that is more difficult to treat. Furthermore, all ablation procedures in our center are performed using robotic magnetic navigation system (RMN); however, the use of the AcQMap mapping system is not limited to RMN, it can be performed during manual CA procedures too. It is remarkable that as compared to historical literature data, our results show superior acute procedural efficacy and much lower documented recurrences during the follow-up. Apparently, the technical limitations of the standard mapping techniques can be responsible for a high recurrence rate among CA-treated patients. Considering the development of cardiac mapping systems, we assume that success rates of CA procedures in AT treatment can be even further increased, and the recurrence rates can be decreased using novel mapping methods.

### Study limitations

The major limitation of this study as first sight is the limited number of patients. Although, the number of patients is only slightly more than 10% of the screened patients, one should note that it is still a unique patient population that was never described before in the context of successful ablation. Indeed, we reached a success rate of more than 90% in a population in which previously CA was not possible. Although, the included study groups are heterogeneous, we performed in-depth analysis for each study group (*de novo* and redo). Another issue is the retrospective nature of the study and the lack of control group. Obviously, it would be interesting to perform a prospective study comparing current methods using AcQMap, but it may not be plausible giving the fact that expected success rate using conventional mapping is too low, and this would raise ethical issues based on our current results. It is important to note that the application number reported in our manuscript may seem excessive; however, for redo patients, it was in some cases necessary to (re)isolate the PVs; this might have caused the increased number of applications. On top of that, reentry-mechanisms require additional ablation lines during the procedures; this also increases the application number. Furthermore, the German registry was the only available report in the literature with reliable data on mapping times we could compare our data to. Although mapping times were remarkably shorter compared to previously reported data, it is notable that neither total procedure duration nor total radiation doses were reduced with the current ablation technique. This can be explained by the unique nature of this patient population, which was not accepted for ablation before.

## Conclusion

AT represents an important cause of SVTs. Mapping brief episodes of ATs and PACs was one of the most challenging aspects in the diagnosis and treatment of symptomatic patients. The AcQMap mapping system provides new possibilities in mapping brief episodes of ATs and PACs, which were previously unmappable using contact-based EAMS or other noncontact mapping modalities. We documented a high procedural success rate (96.8%) and a favorable recurrence rate (19.4%) in patients with ATs mapped with the AcQMap system.

## Supplementary Information

Below is the link to the electronic supplementary material.

Supplementary file1 (MP4 129 KB) Standard single color video showing focal PAC originating in the left atrium (described in Figure 1)

Supplementary file2 (MP4 3250 KB) Standard single color video showing macro-reentry AT around the LAA (described in Figure 2)

## Data Availability

The data underlying this article will be shared on reasonable request to the corresponding author.
